# Growth of children with biliary atresia living with native livers: impact of corticoid therapy after portoenterostomy

**DOI:** 10.1007/s00431-018-3302-z

**Published:** 2018-12-05

**Authors:** Satu Maria Ruuska, Mitja Tapani Lääperi, Maria Hukkinen, Hannu Jalanko, Kaija-Leena Kolho, Mikko P. Pakarinen

**Affiliations:** 1 Department of Gastroenterology, Children’s Hospital, Helsinki University Hospital, PL 347, 00029 HUS Helsinki, Finland; 2 Pediatric Liver and Gut Research Group, Children’s Hospital, Helsinki University Hospital, Helsinki, Finland; 3 Pediatric Research Center, Children’s Hospital, Helsinki University Hospital, Helsinki, Finland; 4 Section of Pediatric Surgery, Children’s Hospital, Helsinki University Hospital, Helsinki, Finland; 5 Department of Pediatric Nephrology and Transplantation, Children’s Hospital, Helsinki University Hospital, Helsinki, Finland; 6 University of Tampere, Tampere, Finland; 7 Tampere University Hospital, Tampere, Finland

**Keywords:** Age and sex-adjusted body mass index (ISO-BMI), Biliary atresia (BA), Corticosteroids, Portoenterostomy

## Abstract

We addressed growth of biliary atresia (BA) patients living with native livers between ages 0–6 and effects of post-surgical corticosteroid treatment on growth. Growth charts of 28 BA patients born in Finland between 1987 and 2017 were retrospectively evaluated. Dosage and length of corticosteroid treatment and hydrocortisone substitution were reviewed. At birth, BA patients were shorter (median height − 0.6 (interquartile range (IQR) − 1.3 to − 0.1) SDS, *n* = 28, *P* < 0.001) than general population. Height remained stable during early childhood (median height − 0.6 (IQR − 1.4 to 0.1) SDS for girls and − 0.4 (IQR − 1.6 to 0.2) SDS for boys at 6 years of age). Patients were of normal height adjusted weight at 6 years with a median age and sex-adjusted body mass index (ISO-BMI) of 20.9 (IQR 19.3 to 25.0) for girls and 22.1 (IQR 20.7 to 25.6) for boys. Higher (≥ 50 mg/kg) cumulative post-portoenterostomy prednisolone dosage resulted in 0.18 SDS lower height per treatment week (*β* − 0.18, SE 0.04, *P* < 0.001) compared to lower dosage (< 50 mg/kg).

*Conclusion*: BA patients grow normally during early childhood. As high postoperative corticosteroid dosage has a short-term negative effect on height, very high dosages should be avoided.

**Table:** 

**What Is Known:**	
***•*** *Growth of biliary atresia patients has mostly been shown to be within normal limits*	
*• Corticosteroids may decrease growth rate*	
**What Is New:**	
***•*** *Biliary atresia patients surviving with their native livers are shorter than general population and their mid-parental target height at birth*	
*• A high (> 50 mg/kg) cumulative prednisolone dosage has a negative transitory impact on height gain after portoenterostomy*

## Introduction

Biliary atresia (BA) is an idiopathic fibro-obstructive cholangiopathy manifesting in infancy [20]*.* The incidence of BA is varied between 1:8000 and 1:10000 reported in Asia to 1:17000–1:20000 in Northern Europe [26–28]. Untreated, obstruction of intra- and extrahepatic biliary tracks leads to fibrosis, liver failure, and death in the first 2 years. The current first-line surgical treatment is portoenterostomy (PE). Provided PE restores adequate bile flow, between 23% and 44% of patients survive with native livers until the age of 20 [10, 29, 41]. Liver transplantation (LT) remains a second-line treatment option and BA is the most common indication for childhood LTs [20, 30].

Following PE, commonly used adjunct postoperative management includes ursodeoxycholic acid, prophylactic antibiotic treatment, and nutritional management [11, 47, 49]. Corticosteroids may improve clearance of jaundice (COJ) rates but potential side effects include an elevated risk for infections, gastrointestinal bleeding, osteoporosis, and poor growth [5, 7, 9]. Growth patterns of patients surviving with their native livers have rarely been reported on, and data on the effect post-PE corticosteroids have on growth are very limited [1, 5, 12, 34]. The aim of this study was to characterize growth of BA patients surviving with their native livers between the ages 0–6. Furthermore, we measured the effect of post-PE corticosteroids on growth.

## Methods

### Patients

We included term BA patients born in Finland between 1987 and 2017 who had normalized their bilirubin < 20 μmol/l after PE, were at least 4 months old, and were living with their native livers as of 31st of December 2017. Data on gestational age, birth height, and weight, associated congenital malformations, BA-type [36], age at surgery, serial height and weight measurements, corticosteroid treatment, and subsequent hydrocortisone substitution were retrospectively collected from medical records. Term infant was defined as weight at or above 2500 g and gestational age between 37 and 42 weeks at birth. Biliary atresia splenic malformation (BASM) was defined as presence of poly- or asplenia [8]. Clearance of jaundice was defined as serum total bilirubin < 20 μmol/L.

### Growth data

Height and weight at birth as well as at 3, 6, 9, 12, 15, 18, 21, and 24 months and at yearly intervals between 2 and 20 years of age were retrospectively collected from medical records. Height and weight at PE and at 1, 2, 3, 4, 5, 6, 9, 12, 15, 18, 21, and 24 months after PE were also retrieved from medical records. For the first year, measurements performed within a ± 4-week period of the exact time point were accepted but for growth data after portoenterostomy, for the first 12 months, the nearest available measurement was accepted if it was within a ± 2-week period of the exact time point. After 12 months, measurements within a ± 6-week period of the except time point were accepted.

Height was analyzed as standard deviation scores (SDS) from the mean of age- and sex-adjusted national values [39]. Normal height was defined as height SDS between − 2.0 and 2.0. Mid-parental target height SDS were calculated according to the equations: 0.791 × mean parental height SDS − 0.147 for girls and 0.886 × mean parental height SDS – 0.071 for boys [40]. Between birth and 2 years of age, relative weight was analyzed as weight-for-length, i.e., the percentage deviation of weight from the median weight for length and sex (DW%) [39, 44]. After the age of 2, relative weight was analyzed with age- and sex-adjusted body mass index (ISO-BMI) [39]. During the first 2 years, underweight was defined as DW% < − 20.0, normal weight as DW% between − 20.0 and + 20.0 and overweight as DW% > 20.0 [39, 44]. For relative weight between ages 2–6, the following ISO-BMI categories were used: underweight (< 17 kg/m^2^), normal weight (17–25 kg/m^2^), overweight (> 25 kg/ m^2^), and obesity (> 30 kg/m^2^) [39]. While calculating changes in relative weight after portoenterostomy, relative weight was analyzed as weight-for-length for consistency. The most recently published national reference data was used for calculation of SDS of weight and length [39].

### Corticosteroid treatment

Cumulative dosage of glucocorticoid treatment after PE, the length and dosage of subsequent hydrocortisone substitution regimen, and measured serum cortisol values after corticosteroid treatment were reviewed from patient records. Corticosteroid dosage was converted to equivalents of prednisolone, and total cumulative dosage of prednisolone (mg) per weight (kg) was calculated. In converting, 5 mg of prednisolone was considered to be equivalent to 5 mg of prednisone, 20 mg of hydrocortisone, 670 μg of dexamethasone, and 4 mg of methylprednisolone [31]. A high total prednisolone dosage was considered to be ≥50 mg/kg [9]. All possible glucocorticoid usages related or unrelated to BA after the initial treatment period were recorded. The relationship of cumulative glucocorticoid dosage to changes in relative height and weight 2 years after PE were analyzed. While analyzing the impact corticosteroids had on growth, possible impact was hypothesized to start at the beginning of medication and continue until 24 months. Prior to national centralization of BA treatment in 2005 [26], varied postoperative management protocols were used with a median cumulative postsurgical corticosteroid dosage corresponding to 50 mg/kg (IQR 26 to 62) of prednisolone. After centralization, adjuvant corticosteroid therapy after PE has been used [22]. Between 2005 and 2014, corticosteroid therapy with oral dexamethasone corresponded to cumulative dosage of 45 mg/kg of prednisolone; since 2015, the dosage has corresponded to 75 mg/kg of prednisolone.

### Statistical methods

Values are expressed as medians with interquartile range (IQR) unless otherwise stated. Wilcoxon signed-rank test was used to compare matched groups. Growth of patients was modeled using linear mixed models [4]. Models included random effect of participants on all time terms. Analyses were carried out in R version 3.4.1 using packages lme4 and lmerTest [25, 38]. Degrees of freedom were estimated using Satterthwaite approximations. The statistically significant level was set to *P* < 0.05.

## Results

### Patient characteristics

During the study period, 96 patients were diagnosed with BA in Finland. Of these, 30 patients had died, 28 were alive with a liver transplant (median age at transplantation 1.45 (0.84 to 2.51) years), 3 were born preterm and 1 had a birthweight < 2500 g. Thus, 34 patients fulfilled the initial inclusion criteria. Six patients were excluded as follows: two because of missing height measurements at birth and four because both parents were non-European. As shown in Table 1, median gestational age was 39 weeks. Most patients were diagnosed with BA type 3 and PE was performed at median age 63 days. Median follow-up age was 8.1 years and patients had well-preserved liver function.

**Table 1:** Patient characteristics

	All patients	Patients with glucocorticoid data
Number of patients	28	24
Gestational age, wk, median (IQR)	39 (38–40)	39 (38–40)
Females, *n* (%)	15 (54)	12 (50)
Birth weight, median (IQR)		
kg	3.340 (3.081–3.669)	3.363 (3.081–3.669)
%	0.0 (− 5.0–5.5)	0.0 (− 6.5–5.5)
Birth height, median (IQR)		
cm	50.0 (48.6–51.0)	50.0 (49.0–51.0)
SD	− 0.6 (− 2.2–(− 0.1))	− 0.6 (− 1.0–0.3)
Biliary atresia type *n* (%)		
1	0 (0.0)	0 (0.0)
2	2 (7.1)	0 (0.0)
3	26 (92.9)	24 (100)
Biliary atresia splenic malformation, *n* (%)	3 (11)	3 (13)
Any other anomaly, *n* (%)	10 (36)	8 (33)
Age at portoenterostomy (PE), days, median (IQR)	63 (24–83)	65 (26–90)
Time to clearance of jaundice, months, median (IQR)	2 (1–3)	2.5 (1–3.8)
Diagnosed with portal hypertension, *n* (%)	12 (43)	12 (50)
Age at last follow-up, years, median (range)	8.1 (0.3–19.7)	7.8 (0.3–19.7)
Biochemical markers at last follow-up:		
Bilirubin, μmol/L, median (IQR)	8.5 (7–14)	8.5 (5.5–14.8)
Conjugated bilirubin, μmol/L, median (IQR)	4 (3–7)	4.0 (3–7.8)
Alanine aminotransferase, U/L, median (IQR)	40 (19–76)	55 (27–77)
Aspartate aminotransferase, U/L, median (IQR)	54 (34–90)	57 (42–100)
Glutamyl transferase, U/L, median (IQR)	48 (21–100)	65 (27–105)
Albumin, g/L, median (IQR)	38 (32–40)	37 (32–40)
Thromboplastin time %, median (IQR)	87 (73–100)	87 (73–105)
Thrombocytes, × 10^9^/L, median (IQR)	174 (79–268)	155 (71–268)

*IQR* interquartile range, *PE* = portoenterostomy. Biliary atresia types as defined by Ohi et al. [18], portal hypertension as defined by Shneider et al. [42]

### Height gain between 0 and 6 years

At birth, BA patients were shorter (median height − 0.6 (− 1.3 to − 0.1) SDS for all patients, *n* = 28, *P* < 0.001) than the general population. Median height SDS of patients with mid-parental target height (MPH) data (*n* = 20) was − 0.2 (− 1.2 to 0.2), which was 0.1 SDS below their median MPH of − 0.1 (− 0.4 to 0.4) SDS (median difference 0.3, *n* = 20, *P* < 0.05).

**Fig. 1:**
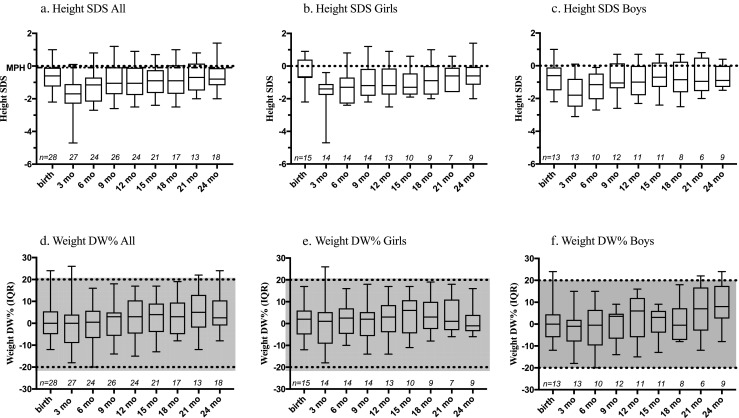
Growth of BA patients during first 2 years, postnatal age, 3-month intervals. **a** Height SDS of all patients. **b** Height SDS of girls. **c** Height SDS of boys. **d** Weight-for-length (DW%) of all patients. **e** Weight-for-length (DW%) of girls. **f** Weight-for-length of boys (DW%). Gray area between dashed lines: normal weight with DW%. Data are presented with median and interquartile range (percentile 25–75). SDS, standard deviation scores from the mean of age- and sex-adjusted value; DW%, percentage deviation of weight from median weight for length and sex; MPH, mid-parental target height

Growth rate appeared to slow down during the first 3 months, as median height for all patients at 3 months was − 1.70 (− 2.3 to − 1.1) SDS. Change in growth rate was more drastic for boys (*n* = 13); their median SDS changed from − 0.6 (− 1.5 to − 0.1) at birth to − 1.8 (− 2.5 to − 0.8) at 3 months while girls’ (*n* = 15) median SDS changed from − 0.7 (− 0.7 to 0.4) SDS to − 1.4 (− 1.8 to − 1.1) SDS (Fig. 1). At 6 months, boys’ growth rate accelerated as median height for all patients was − 1.2 (− 2.2 to − 0.7) SDS, − 1.2 (− 2.1 to − 0.5) SDS for boys and − 1.3 (− 2.3 to − 0.7) SDS for girls. Between 6 and 24 months, growth rate was stable (Fig. 1). At 2 years, patients remained shorter (median height for all patients − 0.8 (− 1.2 to − 0.1) SDS) than the general population. At 2 years, 11/13 (85%) of the patients had height SDS below MPH (median difference = 0.8, *n* = 13, *P* < 0.05).

Height gain was stable between ages 2–6. Girls’ height was unaltered with median height at − 0.6 (− 1.2 to − 0.1 and − 1.4 to 0.1 at 2 and 6 years, respectively) SDS while boys’ median height changed from − 0.9 (− 1.3 to 0.0) SDS to − 0.4 (− 1.6 to 0.2) SDS.

### Weight gain between 0 and 6 years

At birth, patients were of normal weight with median 0.0 (− 5.0 to 5.5) DW% for all, 2.0 (− 5.0 to 6.0) DW% for girls and 0.0 (− 6.0 to 4.5) DW% for boys. During the first 2 years, both genders remained of normal weight with median of 2.5 (− 1.0 to 10.5) DW% for all, − 1.0 (− 3.5 to 4.0) DW% for girls and 8.0 (2.5 to 17.5) DW% for boys at 2 years of age (Fig. 1).

**Fig. 2:**
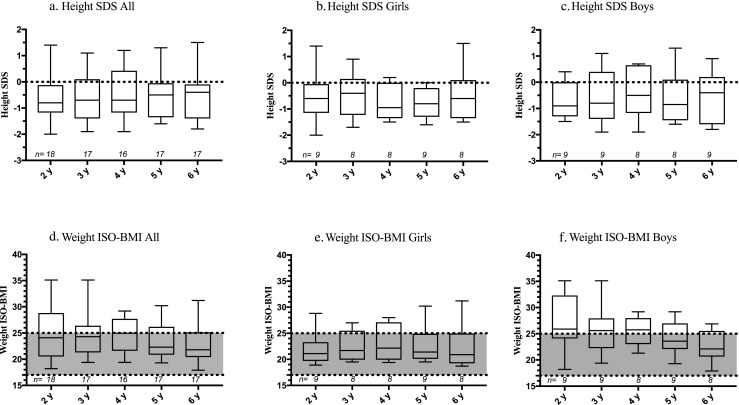
Growth of BA patients between 2 to 6 years with yearly intervals. **a** Height SDS of all patients. **b** Height SDS of girls. **c** Height SDS of boys. **d** Weight ISO-BMI of all patients. **e** Weight ISO-BMI of girls. **f**. Weight ISO-BMI of boys. Gray area between dashed lines: normal weight with ISO-BMI. Data are presented with median and interquartile range (percentile 25–75). SDS, standard deviation scores from the mean of age- and sex-adjusted value; ISO-BMI, age- and sex-adjusted body mass index

When analyzing with ISO-BMI, at 2 years, overall median ISO-BMI was 24.1 (20.5 to 28.8), i.e., on the upper margin of normal range. There was a distinct difference between genders; girls were of normal weight with median of 21.1 (19.7 to 23.3) ISO-BMI while boys were overweight with a median of 25.9 (24.1 to 32.3) ISO-BMI (Fig. 2)*.* Girls’ weight remained constant between ages 2–6 years with a median of 20.9 (19.3 to 25.0) ISO-BMI at 6 years. Boys’ weight decreased slightly with − 1.06 (*β* − 1.06, SE 0.33, *P* < 0.05) ISO-BMI yearly with median ISO-BMI at 22.1 (20.7 to 25.6) at 6 years (Fig. 2)*.*

### Postoperative corticosteroid treatment and growth

Complete data for postsurgical corticosteroid treatment was available for 24 out of 28 patients, including 3 patients not treated with corticosteroids. Corticosteroid treatment started at median 5 (range 4–17) days after PE at median age of 70 (range 18–167) days. The median length of corticosteroid-treatment was 18.5 (range 0–49) days. Sixteen patients received hydrocortisone substitution. Total median cumulative corticosteroid dosage was 277.6 (186.6 to 374.2) mg and 63.6 (47.7 to 74.6) mg/kg of prednisolone excluding hydrocortisone substitution. Median hydrocortisone dosage during the first 30 days of substitution was 8.2 (range 5.0–21.2) mg/m^2^/day and median treatment length was 37 (range 12–133) days. Serum cortisol values were suppressed after glucocorticoid treatment in 12 out of 19 patients (63%) with measured values.

At PE (median age 65 (IQR 26 to 90) days), patients were 1.51 (*β* − 1.51, SE 0.19, *P* < 0.001) SDS shorter than the general population. A growth model for cumulative prednisolone dosage showed 0.16 (*β* − 0.16, SE 0.04, *P* < 0.001) SDS decrease in height for each 100 mg of prednisolone. In a mixed model, there was 0.18 (*β* − 0.18, SE 0.04, *P* < 0.001) SDS decrease in height for each 100 mg of prednisolone while hydrocortisone substitution caused faint 0.07 (*β* 0.07, SE 0.02, *P* < 0.05) SDS increase in height per treatment week. A weight-adjusted dosage model showed negative effect on height with 0.25 (*β* − 0.25, SE 0.05, *P* < 0.001) SDS decrease for each 100 mg of prednisolone per treatment week. In a mixed model, weight-adjusted dosage of prednisolone demonstrated 0.27 (*β* − 0.27, SE 0.05, *P* < 0.001) SDS decrease for each 100 mg of prednisolone per treatment week while hydrocortisone substitution caused 0.07 (*β* 0.07, SE 0.02, *P* < 0.01) SDS increase in height per week. A higher total corticosteroid dosage (> 50 mg/kg) resulted in 0.18 (*β* − 0.18, SE 0.04, *P* < 0.001) SDS lower height per treatment week compared to lower dosage. The effect remained in a model including higher corticosteroid dosage (*β* − 0.21, SE 0.04, *P* < 0.001) and hydrocortisone substitution (*β* − 0.05, SE 0.02, *P* < 0.01). Suppression of cortisol production caused 1.37 (*β* − 1.37, SE 0.55, *P* < 0.05) SDS decrease per week in height compared to unsuppressed patients (Table 2)*.*

**Table 2:** Linear mixed models’ results for height and weight for different parameters. The estimates are for either changes in standardized height (SD) or ISO-BMI. Models for (1.) time only, (2.) time and cumulative prednisolone, (3a.) time and weight-adjusted prednisolone, (4a.) time and high prednisolone dosage, and (5.) time and cortisol suppression. The (b) models extended the (a) models with hydrocortisone substitution

Models for height^a^:	Parameter	Estimate, *β*	Standard error, SE	*P*
1.	Intercept	− 1.51	0.19	*< 0.001*
	Time, 1 month	0.05	0.01	*< 0.001*
2 a.	Cumulative prednisolone per 100 mg	− 0.16	0.04	*< 0.001*
b.	Cumulative prednisolone per 100 mg	− 0.18	0.04	*< 0.001*
	Hydrocortisone substitution per week	0.07	0.02	*0.007*
3 a.	Weight-adjusted prednisolone per 100 mg per week	− 0.25	0.05	*< 0.001*
b.	Weight-adjusted prednisolone per 100 mg per week	− 0.27	0.05	*< 0.001*
	Hydrocortisone substitution per week	0.07	0.02	*0.004*
4 a.	High dosage per week	− 0.18	0.04	*< 0.001*
b.	High dosage per week	− 0.21	0.04	*< 0.001*
	Hydrocortisone substitution per week	− 0.05	0.02	*0.006*
5.	Cortisol suppression per week	− 1.37	0.55	*0.014*
Models for weight^a^:				
1.	Intercept	− 0.44	1.53	0.776
	Time, 1 month	0.31	0.11	*0.011*
2 a.	Cumulative prednisolone per 100 mg	0.58	0.40	0.144
b.	Cumulative prednisolone per 100 mg	0.47	0.42	0.263
	Hydrocortisone substitution per week	0.15	0.17	0.384
3 a.	Weight-adjusted prednisolone per 100 mg per week	0.32	0.47	0.496
b.	Weight-adjusted prednisolone per 100 mg per week	0.16	0.49	0.751
	Hydrocortisone substitution per week	0.19	0.17	0.259
4 a.	High dosage per week	1.12	0.36	*0.002*
b.	High dosage per week	0.87	0.38	*0.024*
	Hydrocortisone substitution per week	0.32	0.15	*0.033*
5.	Cortisol suppression per weeks	2.43	5.15	0.638

^a^All models adjusted for follow-up time, except for the first (1.) time only univariate model

Patients were of normal weight with − 0.44 (*β* − 0.44, SE 1.53, *P* 0.776) DW% at time of portoenterostomy. A growth model for total cumulative prednisolone showed no effect on weight, nor did a growth model combining cumulative prednisolone and hydrocortisone substitution (Table 2*).* A growth model with weight-adjusted corticosteroid dosage showed no effect of prednisolone on weight nor did a model combining weight-adjusted dosage and hydrocortisone substitution (Table 2)*.* A higher total corticosteroid dosage caused 1.12 (*β* 1.12, SE 0.36, *P* < 0.01) DW% increase in weight per treatment week compared to lower dosage, the effect remained (*β* 0.87, SE 0.38, *P* < 0.05) in a mixed model including hydrocortisone substitution (*β* 0.32, SE 0.15, *P* < 0.05). Suppression of cortisol production had no effect (Table 2).

## Discussion

We describe growth patterns of term BA patients living with native livers from birth until early childhood. We found that they are shorter than general population and their mid-parental target height at birth. Their height gain remains constant during infancy and early childhood. BA patients were born of normal weight and remained of normal weight during first 2 years. Girls remained of normal weight between 2 and 6 years whereas boys’ weight, while analyzed with ISO-BMI, slimmed down from overweight to normal weight. A high cumulative corticosteroid treatment dosage had a marked temporary negative impact on height gain.

Results on weight varied at 2 years of age depending whether relative weight was analyzed with DW% or ISO-BMI; when analyzed with DW%, both girls and boys were of normal weight whereas boys were overweight when analyzed with ISO-BMI. This discrepancy likely stems from the fact that there is no known correlation of DW% and ISO-BMI between ages 0 to 2. Single USA-based study comprising 4348 children found poor correlation between weight-for-height and BMI-for-age between ages 2–5. In line with our findings, high percentage (63.4%) of children had lower weight-for-height percentile than BMI-for-age percentile at the same age [16].

Patients in our study are shorter than general population and their mid-parental target height at birth. BA associated with cytomegalovirus infection has been described to have worse clinical outcome with increased mortality [50], although a previous study from Sweden yielded equal clinical results independent of cytomegalovirus status [15]. Symptomatic congenital cytomegalovirus infection may manifest as stunted growth especially affecting birth weight, however, the vast majority of children with congenital cytomegalovirus infection are asymptomatic [6, 13, 33]. As none of the patients in our study were diagnosed with cytomegalovirus infection, it is unlikely that this virus caused the diminished birth height.

Published data assessing growth of nonjaundiced children with BA living with native livers in early childhood are sparse. Sokol et al. [43] found depressed height and weight *z*-scores but normal weight-for-height *z*-scores for 32 BA patients in early childhood. However, as 21 out of 32 patients in their cohort had signs of cirrhosis although patients were clinically stable, it is likely that our study group consists of healthier patients. Karrer et al. [23] reported normal height scores for the majority of 30 patients surviving at least 10 years after portoenterostomy but their results also included transplanted patients. Thirty-five out of 38 nonjaundiced BA patients surviving with native livers at least 10 years were reported to have normal growth by Valayer [46]. Hadzić et al. [19] found normal height and weight *z*-scores at follow-up in 28 BA patients living with native livers, these patients were also considerable older adolescents compared to our cohort (median age 13.4 (range 10.2–22.2) years vs 8.1 (range 0.3–19.7) years in our cohort). Ng et al. [34] reported normal median height and weight *z*-scores for 219 BA patients (median age 9.7 (range 5.1–17.9)) living with native livers at least 5 years after portoenterostomy. Similarly to our findings, Arvay et al. [2] described decreased height-for-age and normal weight-for-age in a group of 10 BA patients living with native livers between ages 1 to 12. Interestingly, they found BMI and weight-for-age *z*-scores inversely correlated with age [2]. In our study, boys weight decreased between ages 2 to 6. While it is possible to argue that lower relative weight might reflect poorer nutritional status, patients in our study demonstrated unaltered height gain and were of normal weight at the end of follow-up suggesting adequate energy and protein intake to sustain growth.

Postnatal corticosteroids have been used for prevention and treatment of chronic lung disease in preterm infants since 1980s [3]. In preterm infants, systemic corticosteroids have been shown to suppress growth [45, 48]. To our best knowledge, there is only one prior study on the effect of postoperative corticosteroid treatment on growth of BA patients [1]. The role of postsurgical corticosteroids remains polemic as previous studies from Asia [24, 32], Europe [9], and the USA [14] have shown higher clearance of jaundice rates for patients treated with corticosteroids, but this beneficial finding was not reciprocated in a large North-American placebo-controlled trial [5]. The START trial [1, 5] recently reported lower than norms *z*-scores for length from portoenterostomy (69 patients) until 24 months of age (35 patients) and for weight from portoenterostomy (70 patients) until 18 months of age (42 patients) for BA patients treated with post-surgical corticosteroids (cumulative prednisolone dosage 116 mg/kg). They also found significant difference between length *z*-scores at 1, 2, and 3 months after portoenterostomy between patient groups treated with corticosteroids or placebo (70 patients in each group) [1]. In line with these findings, in our study, a higher cumulative corticosteroid dosage (> 50 mg/kg) negatively associated with growth by temporarily significantly slowing height gain. In contrast to START trial, we did not observe a long-term negative impact of corticosteroids on weight or height; this is most likely due to the lower dosage of corticosteroids used in our cohort. In our cohort, growth accelerated between 3 and 6 months of age (median age at portoenterostomy for all patients was 63 days) and height gain was stable after that. Although in cholestatic liver disease in early childhood there are multiple factors, such as changes in growth hormone function [17, 21], inadequate resorption of nutrition [35], and elevated energy consumption [18, 37] possibly affecting growth; the impact of corticosteroids was clearly observable in our cohort of children with well-preserved liver function.

Our study has some limitations. Because data were collected retrospectively, we were unable to capture complete growth data for all patients at all time points. Secondly, patients’ nutritional status before PE and energy and protein intake during follow-up could not be assessed reliably.

In conclusion, we found that BA patients surviving with their native livers are shorter than their background population at birth. Their height gain stays stable during early childhood. High post-PE corticosteroid treatment dosage has a negative impact on height gain and an observable positive impact on weight gain compared to lower dosage, but these effects are of short duration. However, as there are conflicting results regarding the beneficial effects of postsurgical corticosteroid treatment for BA patients, it is wise to minimize the possible risks of treatment and to avoid very high corticosteroid dosages.

## Abbreviations

BA: Biliary atresia
BASM: Biliary atresia splenic malformation
COJ: Clearance of jaundice
DW%: Weight-for-length
IQR: Interquartile range
ISO-BMI: Age- and sex-adjusted body mass index
LT: Liver transplantation
MPH: Mid-parental target height
PE: Portoenterostomy
SDS: Standard deviation scores
